# Adsorptive Removal of PFAS from Aqueous Solutions Using GAC, PAC and Ball-Milled Colloidal Activated Carbon: Characterizing Efficiency, Kinetics, and Mechanisms

**DOI:** 10.1021/acsestwater.5c00641

**Published:** 2025-11-03

**Authors:** Mahlet M. Kebede, Md Abdullah Al Masud, Sarah Ortbal, Won Sik Shin, Mesfin M. Mekonnen, T. Prabhakar Clement, Leigh G. Terry

**Affiliations:** † Department of Civil, Construction, and Environmental Engineering, 8063 University of Alabama, Tuscaloosa, Alabama 35487, United States; ‡ School of Architecture, Civil, Environmental and Energy Engineering, 34986 Kyungpook National University, Daegu 41566, Republic of Korea

**Keywords:** adsorption, long-/short-chain PFAS, microporous, mechanisms, competitive adsorption

## Abstract

Per-and polyfluoroalkyl substances (PFAS) present significant challenges for remediation due to their persistence in nature. Activated carbon is a widely used adsorbent for removing PFAS. In this study, three forms of activated carbon, granular activated carbon (GAC), powdered activated carbon (PAC), and ball-milled colloidal activated carbon (CAC_BM_), are compared for their effectiveness in removing short and long-chain PFAS. Physical modification through ball-milling process enhanced the adsorptive properties of activated carbon, resulting in smaller particle size (*d*_50_ = 0.318 μm), increased surface area (968.59 m^2^ g^–1^), and improved suspension stability compared to conventional GAC and PAC. Kinetic experiments showed that CAC_BM_ demonstrated superior removal efficiencies of long-chain PFAS (up to 89% for perfluorooctanesulfonic acid (PFOS) and 73% for perfluorooctanoic acid (PFOA)), and moderate removal of short-chain PFAS (55% for perfluorobutanesulfonic acid (PFBS) and 30% for perfluorobutanoic acid (PFBA)). The pseudo-first-order model adequately described adsorption trends; however, the pseudo-second-order model provided a better fit, with intraparticle diffusion identified as the rate-limiting step. Isotherm studies indicated that PFAS adsorption aligned well with the Freundlich model. Competitive adsorption experiments revealed a hierarchical pattern. Overall, the study demonstrates CAC_BM_ as a promising adsorbent for remediation of PFAS-contaminated water systems.

## Graphical abstract

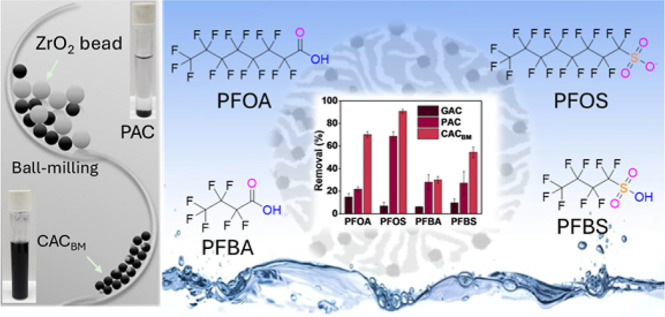

## 1 Introduction

Per- and polyfluoroalkyl substances (PFAS) represent a diverse class of synthetic chemicals that have been extensively utilized in industrial and commercial applications due to their unique chemical properties, including high thermal stability, hydrophobicity, and resistance to degradation.^1–5^ PFAS compounds, used in products such as aqueous film-forming foams (AFFF), nonstick cookware, textiles, and food packaging, have resulted in widespread environmental contamination of soil, groundwater, and surface water.^6–9^ Both long- and short-chain PFAS are of increasing concern due to their persistence, bioaccumulation potential, and association with adverse human health effects including immune system impairment, endocrine disruption, and carcinogenicity.^10,11^ Long-chain PFAS, such as perfluorooctanoic acid (PFOA) and perfluorooctanesulfonate (PFOS), have historically dominated environmental and regulatory discussions.^12^ However, as regulatory actions have led to the phase-out of many long-chain PFAS, the production and environmental presence of short-chain analogues, such as perfluorobutanoic acid (PFBA) and perfluorobutanesulfonic acid (PFBS), have increased.^13,14^ Short-chain PFAS, while less bioaccumulative, are more mobile and harder to remove by conventional treatment technologies, thus presenting new challenges for environmental remediation efforts.^15^

The remediation of PFAS-contaminated matrices poses significant challenges due to the exceptional chemical and thermal stability of these compounds, which are primarily attributed to their strong carbon–fluorine bonds.^16^ Adsorption-based approaches are widely adopted for their simplicity and ability to remove a broad range of PFAS.^12,15,17^ Degradation technologies such as thermal degradation,^18^ plasma reactor,^19^ advanced oxidation processes,^20^ and electrochemical oxidation^21^ can mineralize PFAS and offer long-term solutions, particularly for managing PFAS-laden residuals. Adsorption and destruction technologies have distinct advantages and may be used complementarily depending on treatment goals and site-specific conditions.

Activated carbon (AC) is widely used for PFAS remediation due to its high surface area, hydrophobicity, and ability to adsorb a broad range of organic contaminants through van der Waals forces, hydrophobic interactions, and electrostatic attraction.^22–24^ However, activated carbon performance can vary significantly depending on its physical form, with granular activated carbon (GAC), powdered activated carbon (PAC), and colloidal activated carbon (CAC) exhibiting distinct adsorption behaviors.^25,26^

Colloidal and nanosized activated carbon particles, produced through physical modification process, such as the ball-milling (BM) method, are observed to exhibit enhanced adsorption capabilities that are attributed to increased surface area, reduced diffusion path length, and greater access to internal micropores.^27,28^ Ball-milled colloidal activated carbon (CAC_BM_) has also been found to facilitate more uniform dispersion within porous media or aqueous systems, thereby improving contact efficiency with PFAS molecules and enabling more effective treatment at lower dosages.^29^ Physical modification through BM not only reduces particle size but also introduces surface defects and increases edge-plane exposure, which can enhance PFAS adsorption through both physical and chemical interactions.^30^

Additionally, the use of fine particle suspensions, such as CAC, has gained interest for *in-situ* groundwater remediation applications.^31–34^ The small size and enhanced mobility of colloidal and nanosized activated carbon particles could help facilitate improved subsurface distribution of the particles, resulting in increased contact with PFAS plumes.^35^ However, previous studies have also cautioned that the subsurface transport behavior of fine particles is highly sensitive to other factors such as particle stabilization, density differences, and aquifer heterogeneity.^36,37^ In particular, poorly stabilized colloidal particles may rapidly aggregate and be immobilized near the injection site, reducing aquifer permeability. On the other hand, highly stabilized colloids could behave like a conservative tracer, reducing treatment efficiency. Therefore, a careful assessment of how particle size and other environmental factors (such as ionic strength, pH and NOM) influence the performance of CAC for PFAS removal is essential for optimizing the use of this technology for *in-situ* remediation.

Recent studies have explored ball-milling as a versatile approach for PFAS remediation, either by enhancing the adsorption properties of activated carbon or enabling direct degradation. For example, Yang et al. (2023) demonstrated that piezoelectric ball-milling with boron nitride can effectively destroy PFAS in solid matrices at ambient conditions.^38^ Pilot-scale applications combining cyclodextrin polymers with ball-milling have also achieved high PFAS removal and degradation efficiencies in wastewater treatment.^39^ Parallel to these developments, recent evaluations of two commercially available CAC products, PlumeStop and Intraplex have demonstrated high PFAS removal from aqueous solutions.^35,40^ While PlumeStop contains polymeric stabilizers and Intraplex is polymer-free but involves proprietary modifications,^41^ the undisclosed manufacturing processes make it difficult to isolate the specific role of particle size reduction in PFAS removal. Researchers, however, have highlighted the need to extend these studies by exploring other CAC materials that could be easily synthesized.^35^ This study contributes to the growing body of PFAS remediation research by providing a direct, side-by-side comparison of GAC, PAC, and ball-milled CAC under controlled laboratory conditions, with a focus on adsorption kinetics and the influence of water chemistry. While previous studies (Table S1) have explored PFAS adsorption mechanisms with CAC and the effects of ball-milling with other contaminants, our findings highlight the practical relevance of enhanced kinetics in ball-milled CAC. These insights are particularly valuable for water treatment systems that treat high influent PFAS concentration, such as AFFF impacted sites, membrane concentrates and industrial wastewaters etc., where contact time is limited, and kinetic performance is critical.

In this study, we utilized a simple ball-milling process to synthesize CAC from a commonly used granular activated carbon (GAC). The primary objectives of this study are (1) to evaluate the effectiveness of ball-milled colloidal activated carbon (CAC_BM_) for removing long- and short-chain PFAS from aqueous solutions; (2) to investigate the influence of particle size reduction on changing the surface properties and adsorption capacity of GAC, PAC, and CAC_BM_ particles; (3) to assess the impact of environmental factors, including ionic strength, natural organic matter (NOM) and pH, on the PFAS adsorption behavior of CAC_BM_; and (4) to elucidate the adsorption mechanisms governing PFAS interactions with CAC_BM_ surfaces, considering structural differences between long- and short-chain PFAS molecules.

## 2 Materials and Methods

### 2.1 Chemicals

Perfluorooctanoic acid (PFOA, CAS No. 335-67-1; assay ≥96%) and perfluorooctanesulfonic acid (PFOS, CAS No. 1763-23-1; assay ≥98%) were purchased from Sigma-Aldrich (USA), while perfluorobutanoic acid (PFBA, CAS No. 375-22-4; assay ≥98%) and perfluorobutanesulfonic acid (PFBS, CAS No. 375-73-5; assay ≥97%) were obtained from SynQuest Laboratories (Alachua, FL, USA). Isotopically labeled standards were provided by Wellington Laboratories (Guelph, ON, Canada). Methanol (CH_3_OH, 99.9%, Merck, USA), ammonium acetate (LC–MS grade), and solvents, including acetonitrile and isopropyl alcohol (LC–MS grade) were used as mobile phases and were supplied by Fisher Chemical (USA). Chemical solutions were prepared using deionized (DI) water (Millipore Sigma Synergy Ultrapure Water Purification System, Thermo Fisher Scientific, USA). All experimental reagents were of analytical grade and used without further purification . Humic acid (HA, CAS No. 1415-93-6) was purchased from Spectrum Chemical (NJ, USA). Sodium chloride (CAS No. 7647-14-5), sodium bicarbonate (CAS No. 144-55-8), sodium sulfate (CAS No. 7757-82-6), and sodium phosphate dibasic anhydrous (CAS No. 7558-79-4) were purchased from VWR Chemicals.

### 2.2 Preparation of Ball-Milled Activated Carbon (CAC_BM_)

Powdered activated carbon (PAC) was prepared by mechanically pulverizing coal-based commercial granular activated carbon (GAC) (Norit GAC 830 W), which is produced through steam activation without any chemical or thermal surface modification. To prepare ball-milled colloidal activated carbon (CAC_BM_), 5 g of powdered activated carbon (PAC) was placed into the pulverizing chamber along with 70 g of zirconia balls (ZrO_2_, 94.8%, ϕ = 1 mm; SL Scilab Korea Co., Ltd., Korea), and milling was performed for 4 h. The milling process was paused every 15 min to manually redistribute the material, ensuring uniform grinding throughout the duration (Figure 1). The resulting CAC_BM_ was then separated from the zirconia balls by filtration through a 600 μm stainless steel sieve. The final samples were stored in amber vials for further adsorption experiments and characterizations. The ball-milling conditions used in this study were optimized for laboratory-scale synthesis. The detailed physicochemical characterization of CAC_BM_ is presented in Text S1 and S2.

**Figure 1:**
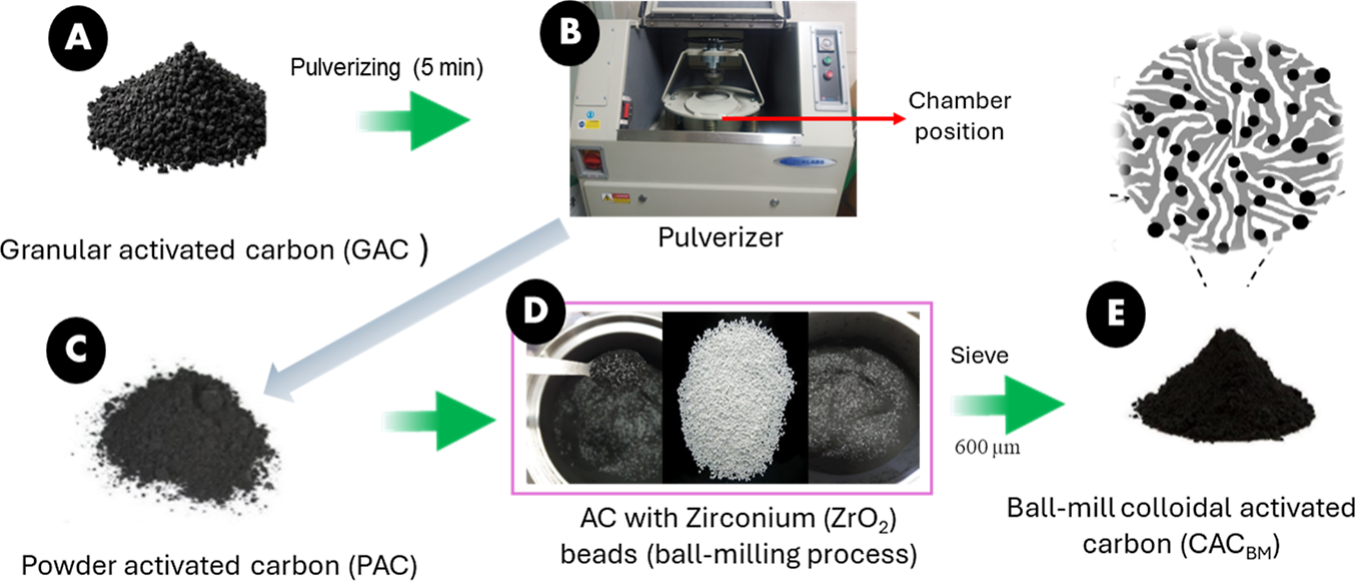
Schematic representation of powder activated carbon (PAC) and ball-milled colloidal activated carbon (CAC_BM_).

### 2.3 Adsorption Experiments and Models

The adsorption experiments were carried out for four PFAS compounds (PFBA, PFBS, PFOA and PFOS) with different chain lengths (C4 and C8) and functional groups (sulfonate or carboxylate) (Table S2). Batch adsorption tests of 1 h duration, isotherm experiments, kinetic studies, pH effect assessment, and selectivity studies were conducted. For each experiment, 150 mL of single solute PFAS solution was added to 250 mL glass Erlenmeyer flasks together with GAC, PAC, or CAC_BM_ at an initial PFAS concentration of 1.0 mg L^–1^. The flasks were placed in a thermostatically controlled shaker at 250 rpm for 1 h. At different time intervals, samples were collected from the flasks for PFAS analysis.

To systematically assess the adsorption performance of CAC_BM_, a series of batch experiments were conducted to examine the effects of key operational parameters, including adsorbent dosage (10–50 mg L^–1^), initial PFAS concentration (0.1–1.0 mg L^–1^), temperature (8–22 °C, where 22 °C represents room temperature), and solution pH (3.0–11.0). Although the tested concentration range (0.1–1.0 mg L^–1^) exceeds current regulatory limits for drinking water, it reflects PFAS levels commonly reported at source zones in AFFF-impacted sites.^34^ This range was selected to better elucidate adsorption mechanisms under high-concentration conditions relevant to contaminated source areas. The pH of the solutions was adjusted using 0.1 M NaOH or HCl. Adsorption selectivity experiments were conducted in the presence of competing background ions using 50 mM solutions of various salts, including NaCl, NaHCO_3_, Na_2_SO_4_, and Na_2_HPO_4_. Furthermore, the effect of NOM was assessed using humic acid (HA) (0 to 30 mg L^–1^).

Isothermal adsorption studies were conducted at ambient temperature over a pH range of 3.0 to 11.0, using initial PFAS concentrations ranging from 0.5 to 12 μM (0.1 to 6.0). An adsorbent dosage of 30 mg L^–1^ was applied for each experiment. The isothermal experiments were carried out in 50 mL centrifuge tubes and agitated in an end-over-end shaker at 25 rpm for 24 h. Following the contact period, samples were filtered using 0.2 μm cellulose acetate syringe filters (VWR). A preliminary evaluation of various syringe filter materials confirmed that cellulose acetate filters exhibited negligible PFAS adsorption and a nondetectable PFAS contamination, ensuring sample integrity.^40,42^

Different kinetic and isothermal models were fitted with experimental data to study the adsorption mechanism of PFAS on CAC_BM_. Three kinetic models, the pseudo-first-order model (PFOM), the pseudo-second-order model (PSOM), and the intraparticle diffusion model (IPDM), and two isotherm models, the Freundlich and Langmuir adsorption models, were investigated (Text S4).

### 2.4 Analytical Method

PFAS was analyzed using Agilent 1290 Infinity II liquid chromatography coupled to 6465A triple quadrupole mass spectrometer system (Ultivo LC/TQ Agilent Inc.). Details on the instrumental operation and quality assurance/quality control (QA/QC), and instrumental conditions can be found in the Supporting Information (Text S5, Tables S3 and S4).

## 3 Results and Discussions

### 3.1 Characterization of CAC_BM_

The surface morphologies of GAC, PAC, and CAC_BM_ samples are presented in Figure 2a–c. The initial rough surfaces of GAC (Figure 2a) and PAC (Figure 2b) were transformed into a smoother surface with homogeneously distributed fine particles following the ball-milling process (Figure 2c). The physicochemical characteristics of GAC, PAC and CAC_BM_ are summarized in Table S5. The BET surface areas (A_BET_) of GAC, PAC, and CAC_BM_ were determined to be approximately 716.51, 729.94, and 968.59 m^2^ g^–1^, respectively (Table S5). The measured BET surface area of GAC (716.51 m^2^ g^–1^) used in this study was lower than the typical values reported for commercial-grade GACs, which are generally around 1000 m^2^ g^–1^.^43–45^ Despite the lower surface area, the GAC served as a relevant baseline for comparison with CAC_BM_, which demonstrated enhanced adsorption kinetics. The surface area, total pore volume, and average pore diameter of CAC_BM_ are 968.59 m^2^ g^–1^, 0.339 cm^3^ g^–1^, 3.32 nm, respectively, which display its excellent adsorption property when compared to GAC and PAC. The CAC_BM_ exhibited a notably higher micropore volume (0.250 cm^3^ g^–1^) compared to its mesopore volume (0.089 cm^3^ g^–1^), as shown in Table S5. This characteristic is particularly significant for the adsorption of PFOA, a long-chain PFAS compound with a molecular size compatible with micropores (<2 nm). Micropores provide a high density of adsorption sites and facilitate strong hydrophobic and van der Waals interactions, which are critical for effective PFOA uptake.^46^ Previous studies have demonstrated that activated carbons with dominant microporosity outperform mesoporous materials in PFAS removal due to enhanced surface area and selective pore accessibility.^47,48^ The superior micropore structure of CAC_BM_ thus contributes to its enhanced adsorption capacity and efficiency in PFAS (i.e., PFOA and PFOS) remediation.

**Figure 2:**
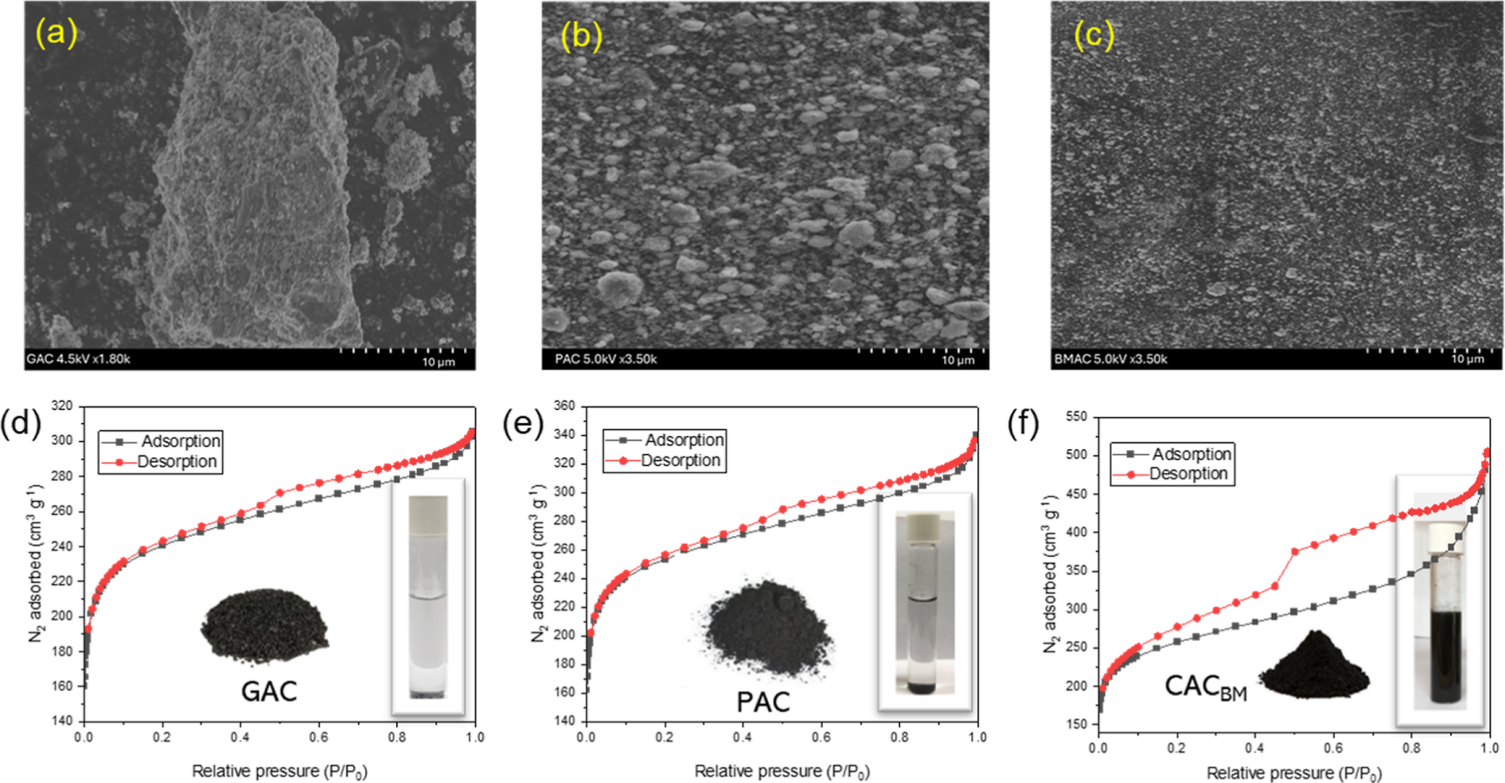
Field emission scanning electron microscope (FE-SEM) images of (a) GAC, (b) PAC, (c) CAC_BM_, (d–f) N_2_ adsorption–desorption isotherms of (d) GAC, (e) PAC, (f) CAC_BM_.

The particle size distribution (*d*_50_) of CAC_BM_ was measured to be 0.318 μm (Table S5). The CAC_BM_ suspension exhibited greater stability in DI water (Figure 2f; inset, after more than 168 h) compared to GAC (Figure 2d; inset, settled within 1 h) and PAC (Figure 2e; inset, settled within 24 h). While stability is expected due to the smaller particle size, the prolonged suspension time offers some advantages for remediation applications. The enhanced dispersion^25,28^ and suspension of CAC_BM_ were found to promote migration, allowing more efficient spatial distribution. A strong linear correlation was observed between pore volume and surface area, indicating that increased pore volume enhances surface area, which may contribute to improved adsorption performance of CAC_BM_.^49^ In contrast, a negative correlation between pore size and surface area was observed, indicating that smaller pore sizes are associated with increased surface area. The higher BET surface area and pore volume of CAC_BM_, attributed to its smaller pore size, may enhance its suitability for surface-interaction-dependent applications such as PFAS capture.^50^ The increase in both pore volume and BET surface area observed after ball-milling is attributed to mechanical fragmentation, exposing previously inaccessible internal pores and creating new porosity through matrix disruption.^51,52^ The particle size reduction from 59.23 to 0.32 μm supports extensive fragmentation, while ultrafine particle aggregation may create interparticle void spaces.^53^ The concurrent decrease in average pore size despite increasing total porosity indicates predominant formation of new mesopores through fragmentation of larger pores and generation of fresh fracture surfaces with high mesopore density.^25,28,51,52^ Although previous studies have reported mesopore collapse during pulverization, our results show increased pore volume and surface area, suggesting that controlled ball-milling conditions can enhance rather than destroy mesoporous structure through pore multiplication. However, further pore structure analysis using DFT or NLDFT methods is warranted to confirm the detailed pore size distribution changes. In addition, although BET-derived parameters are model-dependent and subject to autocorrelation, the consistent trends across replicate measurements suggest a real enhancement in accessible porosity due to ball-milling.

The concentrations of acidic and alkaline sites of CAC_BM_ were determined by pH drift and the Boehm titration method, respectively (Text S3). The result shows that surface chemistry of CAC_BM_ reveals notable modifications compared to PAC, particularly in terms of functional group composition and acid–base properties (Table S6). CAC_BM_ exhibits higher concentrations of carboxyl (50.98 μmol g^–1^) and phenol (64.93 μmol g^–1^) groups, which are known to enhance hydrophilicity and contribute to electrostatic interactions with anionic PFAS species. Despite a slight reduction in lactone content, the overall surface acidity of CAC_BM_ (131.58 μmol g^–1^) is increased relative to PAC (120.60 μmol g^–1^), suggesting a greater density of oxygen-containing functional groups introduced during ball-milling. This increase in acidic sites may facilitate stronger adsorption through hydrogen bonding and dipole interactions. Conversely, the slight decrease in alkalinity (105.39 μmol g^–1^) may reduce competition from basic sites, further favoring PFAS uptake.

### 3.2 Performance of CAC_BM_ under Different Physicochemical Conditions

The adsorption performance of CAC_BM_ was systematically evaluated for the removal of four different PFAS compounds under varying physicochemical conditions. Parameters assessed included adsorbent type and dose, initial PFAS concentration, and pH of solution.

As shown in Figure 3a, the adsorption performance of CAC_BM_ was compared with that of commercial GAC and PAC. While PFAS adsorption onto GAC typically requires extended contact times to reach equilibrium, the 1 h batch experiments were conducted to evaluate initial adsorption kinetics under limited contact conditions, recognizing that longer residence times are typical in field applications. Under these conditions, the comparative performance of GAC, PAC, and CAC_BM_ highlights the enhanced adsorption kinetics of CAC_BM_, particularly for long-chain PFAS. The removal efficiency of PFOA (73% ± 24%) and PFOS (89% ± 35%) is higher than that of PFBA (30% ± 11%) and PFBS (55% ± 18%) in all three adsorbents (Figure 3a), as long-chain PFAS are more adsorbed on carbonaceous-based adsorbents than short-chain PFAS.^54^ CAC_BM_ has a higher removal efficiency for long-chain PFAS than GAC and PAC, while it only shows a slight increase for short-chain PFAS, particularly for PFBA. CAC_BM_ smaller pore size likely resulted in shorter internal diffusion and better accessibility of adsorption sites compared to GAC and PAC.

**Figure 3:**
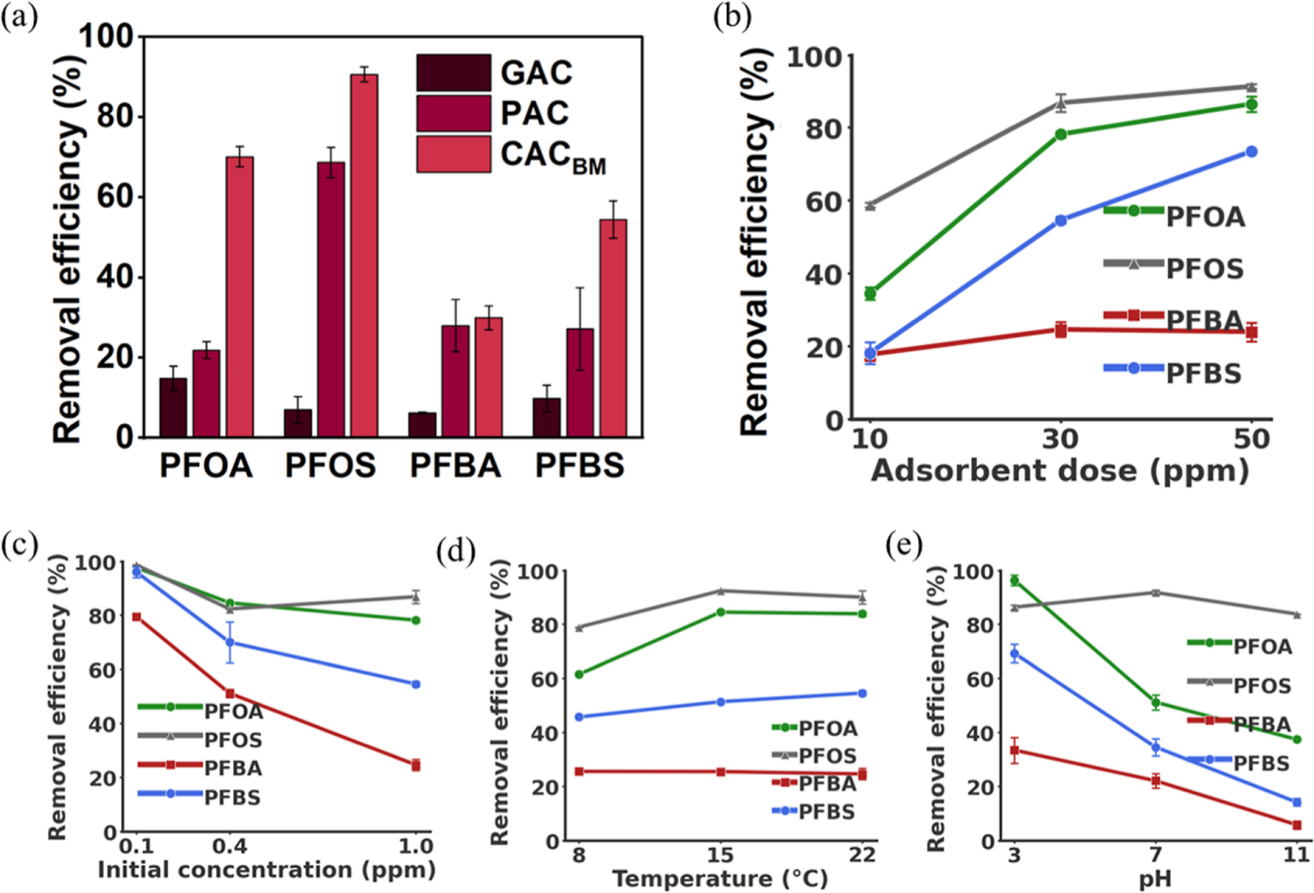
Removal efficiency of PFAS in 1 h batch experiments varying the following properties (a) adsorbent types, (b) CAC_BM_ adsorbent doses, (c) initial PFAS concentrations, (d) temperatures, and (e) pH values. The following experimental conditions were maintained when the primary property was investigated: [PFAS]_0_ = 1.0 mg L^–1^, [CAC_BM_ Adsorbent]_0_ = 30 mg L^–1^, Temperature = 20 °C (room temperature), pH = 6.2 (unadjusted pH).

To investigate the effect of dosages, 10 to 50 ppm of CAC_BM_ was used. PFAS removal efficiencies increased with an increase in adsorbent dosage for all four PFAS (Figure 3b). At 50 ppm, the removal efficiencies for PFOA, PFOS, PFBA, and PFBS reached 87% ± 2%, 91% ± 1%, 24% ± 3%, and 73% ± 1%, respectively. Notably, the increase in removal efficiency was more pronounced for long-chain PFAS (Figure 3b), while the change in PFBA removal remained negligible, increasing only from 18% ± 2% at 10 ppm to 24% ± 3% at 50 ppm, which highlights the challenges of adsorption of PFBA regardless of adsorbent doses. At the 50 ppm dose of CAC_BM_, the adsorption of PFAS was instantaneous, with PFAS rapidly adsorbed within a few minutes. To capture lower values of adsorbed mass per mass of adsorbent in the kinetic study, 30 ppm of adsorbent was used as the optimum dosage for all subsequent experiments.

The removal efficiency of CAC_BM_ decreased as initial PFAS concentrations increased from 0.1 to 1.0 (Figure 3c). PFBA exhibited the most significant decline, with its removal efficiency decreasing from 80% ± 1% to 25% ± 2%. In contrast, long-chain PFAS showed only a slight reduction; PFOA decreased from 98% ± 3% to 78% ± 1%, PFOS from 97% to 87% ± 3%, and PFBS from 96% ± 2% to 55% ± 1%. The highest tested PFAS concentration, 1.0, was used in subsequent experiments.

The adsorption of long-chain PFAS, was slightly lower at 8 °C compared to 15 and 22 °C, although no significant difference was observed between 15 and 22 °C (Figure 3d). Short-chain PFAS showed no significant variation across the tested temperatures. These results indicate that typical groundwater conditions (15 to 20 °C), slightly favor PFAS adsorption on CAC_BM_. This observation remained consistent when adsorption was evaluated at higher temperatures (20–60 °C) (Figure S3).

The maximum removals of PFOA, PFBA, and PFBS were observed at pH 3.0, while PFOS exhibited the highest removal at pH 6.2 (unadjusted pH), as shown in Figure 3e. However, the adsorbed mass of all PFAS decreased significantly as the pH increased from 7.0 to 11.0, with the most pronounced reductions observed for short-chain PFAS, PFBA and PFBS. The observed results may be attributed to the greater hydrophobicity of long-chain PFAS compared to short-chain analogues, as their adsorption is likely governed by a combination of hydrophobic and electrostatic interactions.^55^ The adsorption of long-chain PFAS is presumed to be predominantly controlled by hydrophobic interactions, whereas the adsorption of short-chain PFAS is considered to depend more on electrostatic interactions.^54,56,57^ Accordingly, a reduction in electrostatic attraction under high pH conditions (see Section 3.6-mechanisms) led to decreased adsorption of short-chain PFAS.^58^

### 3.3 Adsorption Kinetics and Equilibrium Isotherms

Kinetic experiments were conducted to evaluate the time-dependent performance of PFAS adsorption on CAC_BM_. Among the pseudo-first-order and pseudo-second-order models (Text S4), a simple pseudo-first-order kinetic model was able to explain the adsorption trends for all the PFAS compounds (Figure 4a).

**Figure 4:**
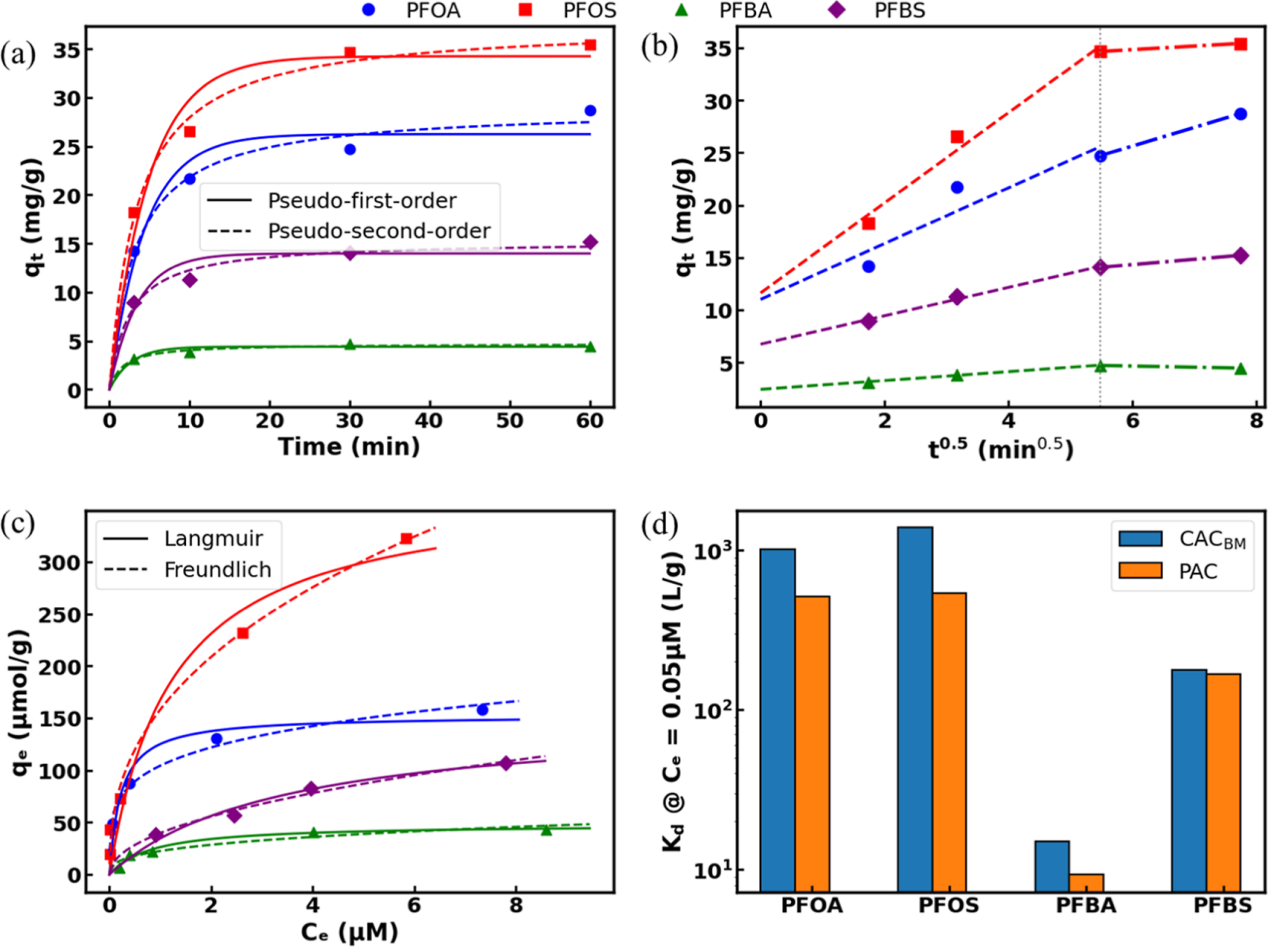
(a) Adsorption kinetic data for PFOA, PFOS, PFBA, and PFBS on CAC_BM_ fitted with pseudo-first and second-order models (initial PFAS concentration was 1.0 mg L^–1^), (b) adsorption kinetic data for PFOA, PFOS, PFBA, and PFBS on CAC_BM_ fitted with intraparticle diffusion model, (c) isothermal adsorption experimental data for PFOA, PFOS, PFBA, and PFBS on CAC_BM_ fitted with Langmuir and Freundlich isotherms, and (d) variation in partitioning coefficient *K*_d_ values at 0.05 μM aqueous concentration for PFOA, PFOS, PFBA, and PFBS on CAC_BM_ and PAC. Experimental conditions: [CAC_BM_ Adsorbent]_0_ = 30 mg L^–1^, pH: 6.2 (unadjusted), Temperature: 22 °C (room temperature). Note: In figure (d), adsorption data of GAC is not provided, as equilibrium may not have been reached within 24 h period (Figure S1).

The rate constant values (*k*_1_, *k*_2_) for CAC_BM_ were found to be higher than those for GAC (Table 1). The faster adsorption kinetics of CAC_BM_ were justified by its significantly reduced particle size, which shortened diffusion pathways to internal adsorption sites and increased external surface area, enhancing the overall mass transfer rate of PFAS. Similar improvement of adsorption kinetics has been highlighted for other organic contaminants that used ball-milled carbon materials in prior studies.^59–62^ In addition, a higher adsorption capacity (*q*_e_), with a more pronounced effect on long-chain PFAS compared to short-chain PFAS, was observed (Table 1). This increased adsorption capacity is likely due to the increased external surface area, enhanced accessibility of adsorption sites, and possible surface oxidation during ball-milling,^63^ which may introduce oxygen-containing functional groups that improve adsorption affinity.

**Table 1:** Kinetic Parameters for PFAS Adsorption on GAC and CAC_BM._

		Pseudo-first-order			Pseudo-second-order			Intraparticle diffusion model (IPDM)				
compound	adsorbent	*k*_1_ (min^–1^)	*q*_e_ (mg g^–1^)	*R* ^2^	*k*_2_ (g mg^–1^ min^–1^)	*q*_e_ (mg g^–1^)	*R* ^2^	*k*_d1_ (mg g^–1^ min^–0.5^)	*C*_1_ (mg g^–1^)	*k*_d2_ (mg g^–1^ min^–0.5^)	*C*_2_ (mg g^–1^)	*R* ^2^
PFOA	GAC	0.11	12.95	0.858	0.01	14.71	0.912	N/A				
	CAC_BM_	0.22	26.23	0.881	0.01	28.96	0.967	2.66	11.02	1.77	14.99	0.865
PFOS	GAC	0.05	21.95	0.994	0.001	29.99	0.998	N/A				
	CAC_BM_	0.20	34.25	0.903	0.01	37.61	0.982	4.29	11.63	0.33	32.79	0.981
PFBA	GAC	0.01	2.42	0.994	0.001	4.34	0.993	N/A				
	CAC_BM_	0.38	4.39	0.783	0.13	4.69	0.919	0.42	2.42	0.01	5.32	0.990
PFBS	GAC	0.03	6.65	0.958	0.003	9.12	0.964	N/A				
	CAC_BM_	0.29	13.98	0.748	0.03	15.27	0.933	1.35	6.75	0.51	11.27	0.993

Analysis of the intraparticle diffusion model results revealed two distinct linear regions (Figure 4b), confirming that multiple processes influence the adsorption kinetics. The first region exhibited steeper slopes (higher *K*_d1_ values) than the second region (lower *K*_d2_ values), indicating that external mass transfer proceeded faster than intraparticle diffusion. The regression lines did not pass through the origin for any scenario (Figure 4b), which confirms that intraparticle diffusion is not the only rate-controlling mechanism.^57,64^ Notably, short chain PFAS exhibited lower *K*_d2_ values, suggesting that their intraparticle diffusion was hindered. This trend aligns with previous reports of chain length dependent diffusion limitation in PFAS adsorption.^65^

In Figure 4c isotherm equilibrium sorption result of q_e_ versus C_e_ is plotted for PFOS, PFOA, PFBS, and PFBA. Experiments were completed on an end-to-end shaker for 24 h at an adsorbent dose of 30 ppm, both temperature and pH unadjusted. As equilibrium solute concentration (*C*_e_) increases adsorbed amounts of PFAS (*q*_e_) increase. The isotherm data were fitted using Langmuir and Freundlich models.^66^ The experimental data were well fitted by both models with *R* ^2^ > 0.922 in all cases (Table 2). Based on the *R* ^2^ values obtained, generally Freundlich model was found to be a better fit, indicating a multilayer adsorption process.^67^ The fitting parameters of both models are listed in Table 2. The Freundlich model parameters (*K*_F_ and *n*) calculated in this study are comparable to those reported for PlumeStop applications, indicating similar adsorption behavior. The observed trend in adsorption capacity (PFBA < PFBS < PFOA < PFOS) aligns with previously published data and supports the conclusion that ball-milled activated carbon exhibits competitive performance relative to CAC studies.^34,35,68^

**Table 2:** Isotherm Study Data Fitting Parameters.

	Langmuir model *q* = *q*_mL_ *b*_L_ *C*/(1 + *b*_L_ *C*)			Freundlich model *q* = *K*_F_ *C* ^1/*n*^		
compound	*q*_mL_ (μmol g^–1^)	*b*_L_ (L μmol^–1^)	R^2^	*K*_F_ ((μmolg^–1^) μmolL^–1^)^−(1/*n*)^	1/*n*	*R* ^2^
PFOA	152.41	4.58	0.93	104.38	0.22	0.98
PFOS	372.50	0.82	0.95	158.19	0.36	0.99
PFBA	48.28	1.20	0.97	22.44	0.34	0.92
PFBS	152.43	0.29	0.97	39.62	0.50	0.98

There is an increase in the adsorption capacity of CAC_BM_ over PAC (Figure 4d). The change is relatively smaller for short-chain PFAS. Compared to CAC_BM_ capacity increases in adsorption kinetics, the change in isothermal data is modest, indicating that ball-milling primarily enhances adsorption rates rather than equilibrium capacity (Figure 4d). The significantly greater effect of ball-milling on adsorption kinetics versus adsorption equilibrium is consistent with previous reports.^59,63^ The results also support the conclusion that physical modification of GAC primarily increases PFAS diffusion into the adsorbent micropores.

### 3.4 Effect of Anions and Humic Acids (HA) on PFAS Adsorption onto CAC_BM_

The presence of background inorganic anions and organic matter in aquatic environments has been recognized as a critical factor influencing the behavior and removal efficiency of target pollutants. The removal efficiencies of PFAS by CAC_BM_ in the presence of 50 mM background anions including Cl^–^, SO_4_^2–^, HCO_3_^–^, and HPO_4_^2–^ are presented in Figure 5a. Overall, short-chain PFAS are highly sensitive to the presence of inorganic anions. In contrast, the adsorption of long-chain PFAS slightly improved in the presence of chloride ions (with removal efficiencies improving by 0.5% to 3%), consistent with previous findings attributing enhanced adsorption at higher ionic strength to ‘salting out’ effect and electric double layer compression, which reduce repulsive electrostatic interactions and promote hydrophobic partitioning.^35,40^ However, both short-chain compounds, PFBA and PFBS, showed decreased adsorptions when in solution with ions for all ions tested, including NaCl. A more pronounced reduction was observed in PFBA, with a decrease adsorption ranging from 50% to 76%, compared to a reduction of 24% to 64% for PFBS. Mole et al. (2024)^40^ similarly found that the presence of 30 mM of NaCl and CaCl_2_ reduced the adsorption coefficient (*K*_d_) of PFBA by over 50%, but the effect on PFBS was not investigated. The observed PFAS reduction in adsorption when in solution with multivalent anions (SO_4_^2–^, HCO_3_^–^, HPO_4_^2–^) can be attributed to either competitive interactions for adsorption sites or surface charge modification.^69^ The background ion experiments highlight that electrostatic interactions govern the adsorption of short-chain PFAS to CAC_BM_, while hydrophobic interaction, enhanced by ionic strength, mainly drives long-chain PFAS adsorption.

**Figure 5:**
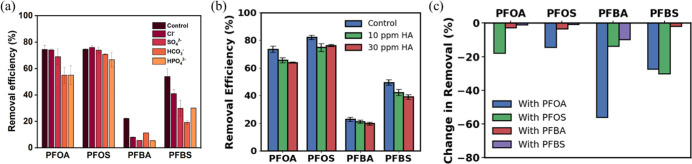
Removal efficiency of PFAS compounds in 1 h batch experiments by varying the following background chemicals: (a) different anions (initial anion concentration is 50 mM), (b) different humic acid concentrations, and (c) effect of binary PFAS mixtures Experimental conditions: [PFAS]_0_ = 1.0 mg L^–1^, [CAC_BM_ Adsorbent]_0_ = 30 mg L^–1^, Temperature = 22 °C (room temperature), pH = 6.2 (unadjusted pH).

The effects of humic acids (Figure 5b) show a slight decrease in PFAS adsorption efficiency as the concentration of humic acids increases. The slight decline is attributed to HA competing for adsorption and partially blocking micropores. Previous studies^35,70^ have similarly reported that low molecular weight organic matters hinder PFAS adsorption, particularly for short-chain compounds, whose uptake relies more heavily on electrostatic interactions and micropore accessibility.

### 3.5 Competitive Adsorption in Binary PFAS Solute Systems

Bisolute experiments were conducted to evaluate how background PFAS mixtures influence competitive adsorption behavior. Figure 5c illustrates the change in removal efficiency (%) between single- and binary-solute systems for various PFAS pairs. Negative values indicate a reduction in removal efficiency in the presence of cocontaminants, suggesting competitive interactions between PFAS compounds. A clear competitive hierarchy was observed based on PFAS chain length and functional group. Short-chain PFAS (PFBA, PFBS) have higher reductions in removal efficiency when in binary solutions with their longer-chain homologues (PFOA, PFOS). The highest reduction in removal efficiency was recorded for PFBA when copresent with PFOA (−56%), PFOS (−13.7%), and PFBS (−9.7%), indicating that short-chain PFAS such as PFBA were readily outcompeted by their longer-chain and more hydrophobic counterparts for available adsorption sites.^58^ These findings are consistent with those reported in a previous competitive adsorption study involving CAC.^29^ Notably, PFOS significantly reduced the removal efficiencies of both PFBA (−13.7%) and PFBS (−30.2%), which is attributed to PFOS’s higher adsorption affinity resulting from its longer carbon chain and sulfonic acid functional group. These structural features enhance hydrophobic interactions and enable PFOS to occupy adsorption sites preferentially. In contrast, competition between PFOA and PFOS was found to be relatively minor. The observed results align with the previous discussion of adsorption mechanisms, i.e., electrostatic interactions, common in short-chain PFAS, are more vulnerable to competitive displacement than hydrophobic interactions that mainly govern long-chain PFAS adsorption.^71^

### 3.6 Adsorption Mechanisms of PFAS

The adsorption behavior of PFAS (PFOA, PFOS, PFBA and PFBS) on carbon-based adsorbents such as CAC_BM_ is governed by a combination of electrostatic interactions, hydrophobic interactions, and surface chemistry of the CAC_BM_. The p*K*_a_ values of the PFAS and the pH_pzc_ of the CAC_BM_ are critical parameters influencing the net charge of both adsorbate and adsorbent, thus affecting their interaction dynamics.

The pH_pzc_ of CAC_BM_ is 5.9 (Figure S4), which implies the surface will be positively charged at pH < 5.9 and negatively charged at pH > 5.9^25,58^. Although reported p*K*_a_ values for PFAS compounds vary considerably across different sources, PFOA is frequently cited with a p*K*_a_ near 2.8. This suggests that under acidic conditions such as pH 3.0 (one of our experimental conditions), PFOA predominantly exists in its anionic form (PFOA^–^) due to partial or complete deprotonation of its carboxylic acid group. Accordingly, at this pH, the electrostatic interaction between the negatively charged PFOA^–^ and the positively charged surface of CAC_BM_ (pHpzc = 5.9) (Figure 6a, zone-2) is expected to enhance adsorption efficiency (Figure S2). At pH values below 2.8, PFOA remains predominantly in its neutral form, while CAC_BM_ carries a positive surface charge; as a result, neither electrostatic attraction nor repulsion is expected (Figure 6a, zone-1). As the pH increases (pH 7.0–11.0), more PFOA molecules convert to their anionic form (PFOA̅), and the surface of the CAC_BM_ becomes increasingly negative since pH will rise above the pHpzc. The negative charge on both the CAC_BM_ surface and the PFOA̅ ions leads to electrostatic repulsion (Figure 6a, zone-3), which should reduce adsorption. Furthermore, the presence of other ions in solution at higher pH may compete with PFOA^–^ for adsorption sites, further decreasing PFOA adsorption (Figure S2).

**Figure 6:**
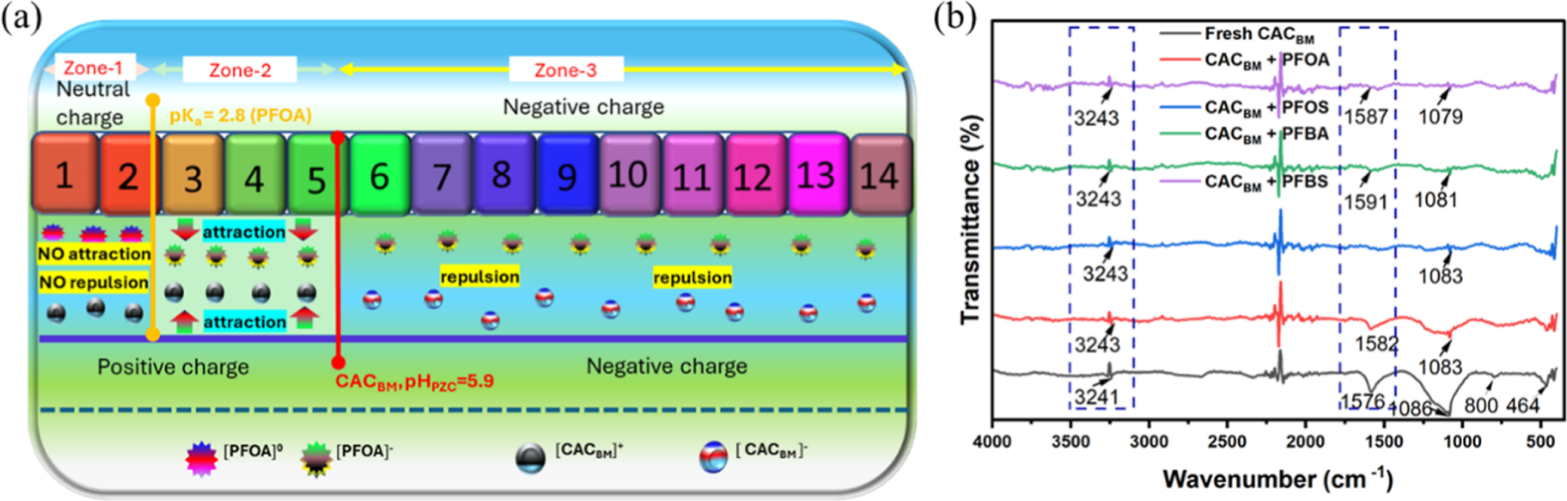
(a) The surface charge mechanism of PFOA and CAC_BM_ under different pH conditions,and (b) FTIR spectra of CAC_BM_ before and after adsorption with single solute PFOA, PFOS, PFBA and PFBS solution.

PFOS has a much lower p*K*_a_ (typically <1), implying that it exists in its anionic sulfonate form (PFOS^–^) across a wide pH range, including at pH 6.0. Despite the CAC_BM_ surface being nearly neutral or slightly negatively charged at pH 6.0 (around its pHpzc = 5.9), PFOS adsorption is still maximized, which suggests that hydrophobic interactions and micelle-like aggregation behavior at which PFOS concentration play a more dominant role than electrostatics in its adsorption.^55^ The longer perfluorinated tail and sulfonic acid group of PFOS enhance its hydrophobicity and adsorption affinity to the carbon surface, especially under weakly acidic to neutral conditions.

PFBA and PFBS were also found to exhibit similar adsorption trends to those observed for PFOA, as shown in Figure S2a–d. The maximum adsorption of PFBA and PFBS at pH 3.0 was primarily attributed to strong electrostatic attraction between their anionic forms and the positively charged surface of CAC_BM_. The adsorbent behavior of the four compounds onto CAC_BM_ is summarized in Table S7.

In addition, the adsorption capacities of short-chain PFAS such as PFBA and PFBS were found to be significantly lower than those of long-chain PFAS such as PFOA and PFOS. This difference can be attributed to the greater number of −CF_2_– units in long-chain PFAS, which confer higher hydrophobicity and promote enhanced partitioning onto the hydrophobic surface of the carbon-based adsorbent.^72,73^ In contrast, short-chain PFAS contain fewer fluorinated carbon atoms, resulting in reduced hydrophobic interactions and, consequently, lower sorption affinity.^74^ This chain length-dependent adsorption behavior is quantitatively supported by the strong correlation (*r* ^2^ = 0.97) observed between the sorption coefficient (*K*_F_) and the octanol–water partition coefficient (log *K*_ow_) of the studied PFAS compounds (Figure S5).

In addition to pH and PFAS molecular structure, other environmental factors ionic strength (Figure 5a), and humic acid (HA) as a part of NOM (Figure 5b) can influence adsorption mechanisms. In our study, elevated ionic strength inhibited PFAS adsorption by CAC_BM_, likely due to competitive interactions between PFAS and background anions for adsorption sites. The presence of high concentrations of anions may also disrupt electrostatic attraction between the negatively charged PFAS molecules and positively charged surface sites, thereby reducing overall adsorption efficiency (Figure 5a). The presence of HA can compete with PFAS for adsorption sites or modify the surface chemistry of CAC_BM_, potentially inhibiting adsorption (Figure 5b). These factors collectively impact colloidal stability and interaction forces and should be considered when evaluating PFAS removal performance under realistic environmental conditions.

Furthermore, the contribution of the micropore-filling phenomenon to the PFAS adsorption process onto CAC_BM_ was evaluated using the Polanyi–Dubinin–Manes (PDM) isotherm model^75^ (Text S4). The adsorption capacity (*q*_max_) of PFAS on CAC_BM_ was found to be higher than that on GAC (Table S8), highlighting that PFAS adsorption onto CAC_BM_ involves mixed mechanisms, including a prominent pore-filling process.

### 3.7 FTIR Evidence of PFAS Adsorption Pathways on CAC_BM_

Fourier-transform infrared spectroscopy (FTIR) analysis of CAC_BM_ was conducted before and after adsorption to identify potential interaction mechanisms between the adsorbent surface and the four PFAS studied. Prior to FTIR analysis, CAC_BM_ samples were oven-dried at 60 °C for 48 h to minimize interference from adsorbed water. The observed spectral bands correspond to characteristic functional groups of activated carbon and are distinct from water-related absorption features. Noticeable spectral changes were observed that provide insights into the adsorption mechanisms involved (Figure 6b).

The FTIR spectra of the fresh CAC_BM_ showed a peak at 3241 cm^–1^, which relates to OH stretching vibrations, indicating that there are hydrophilic parts on the carbon surface that can bond with PFAS compounds.^76^

A clear peak at 1576 cm^–1^, which relates to C=C stretching vibrations in aromatic structures, was seen and shows that there are graphitic structure in the activated carbon, acting as main spots for hydrophobic interactions with the fluorinated alkyl chains of PFAS compounds.^77^ The broadness of the peak at 1576 cm^–1^ indicates a heterogeneous distribution of aromatic structures with varying degrees of conjugation.^78^

The broad peak at 1086 cm^–1^ can be assigned to C–O stretching vibrations in various oxygen-containing functional groups, including alcohols, ethers, and carboxylic acids.^79^ The notable breadth of this peak suggests a diverse range of C–O containing functionalities on the carbon surface that can likely engage in electrostatic interactions with the polar head groups of PFAS.

In addition, the FTIR peaks observed at approximately 800 cm^–1^ and 464 cm^–1^ were attributed to out-of-plane bending vibrations of aromatic C–H bonds or lattice vibrations, which are commonly associated with the carbon backbone and potentially with residual mineral components.

Based on the spectral changes observed after adsorption (Figure 6b), several mechanisms can be proposed for PFAS adsorption. The change and decrease in strength of the aromatic C=C stretching area (1576 cm^–1^) suggest that the fluorinated alkyl chains of PFAS are adsorbing to the graphitic parts of the activated carbon through hydrophobic interactions.^80^ These interactions are typically strong and contribute significantly to the overall adsorption capacity. The changes in the C–O stretching area (1086 cm^–1^) indicate that there are attractive forces between the negatively charged parts of PFAS and the positively charged areas on CAC_BM_.^81^ These interactions are particularly important for the binding of the polar head groups of PFAS compounds. The small change in the O–H stretching band (from 3241 to 3243 cm^–1^) shows that hydrogen bonding occurs between PFAS molecules and hydroxyl groups (−OH) on the carbon surface, which helps increase the overall binding strength.^82^ Overall, the FTIR data highlighted PFAS adsorption potential of CAC_BM_. Furthermore, the adsorption can be attributed to CAC_BM_ having higher aromaticity and to the availability of different surface functional groups (Table S9).

## 4 Conclusions

This study investigated the effectiveness of CAC_BM_ for removing PFAS analytes and compared them with GAC and PAC performance. The results show that the ball-milling process is effective in producing stable colloidal particles with noticeably higher surface area, reduced particle size, and enhanced pore accessibility, which collectively contributed to improved colloidal stability.

Our batch experimental data show that the removal efficiencies for PFOS, PFOA, PFBS and PFBA are 89%, 73%, 55% and 30%, respectively. Kinetic experimental data indicated that while the pseudo-first-order kinetic model adequately described PFAS adsorption trends, the pseudo-second-order kinetic provided slightly better fits. Furthermore, the intraparticle diffusion process appeared to play a role in the overall adsorption process, underscoring the potential benefits of reducing particle size reduction to enhance PFAS uptake.

Varying the background levels of various interfering chemicals, including Cl^–^, SO_4_^2–^, HCO_3_^–^, and HPO_4_^2–^, and humic acid showed that the long-chain PFAS maintained strong adsorption even in the presence of increased background ions. The short-chain PFAS, however, showed reduced adsorption in the presence of competing anions. The competitive adsorption experiments showed that long-chain PFAS outcompeted their short-chain analogues, with PFBA experiencing the most significant displacement.

Our results show that CAC_BM_ is a potential adsorbent that can be used to develop *in-situ* remediation barriers for treating PFAS plumes. However, further studies are needed to understand the transport characteristics of CAC_BM,_ which can be sensitive to various factors including aggregation kinetics, time-dependent settling characteristics, density differences, and aquifer heterogeneity. Furthermore, the effects of cocontaminants in complex mixtures, such as aqueous film-forming foams (AFFF), should also be systematically studied. Additionally, this study utilized a single type of activated carbon (coal-based Norit GAC 830 W), which may limit the generalizability of the findings, as PFAS adsorption performance can vary significantly depending on the precursor type and surface characteristics of the activated carbon (e.g., coconut shell-based AC)^83,84^ Furthermore, scaling up the ball-milling process for full-scale production needs further investigation to assess practical challenges related to energy consumption. A life cycle assessment is necessary to determine whether the observed improvements in sorption performance justify the energy input and production expenses at industrial scales.

## Supplementary Material



## Notes

Physicochemical characterization of CAC_BM_, determination of point of zero charge, adsorption kinetics and isotherm models, PFAS analysis methods, brief summary of previous studies on colloidal activated carbon, physicochemical properties of and pH dependent adsorption behavior of PFAS used in this study, Polanyi-Dubinin–Manes isotherm model parameters, FTIR additional interpretations, additional kinetics data and fitting (i) with extended time up to 24 h, (ii) at different pH values, adsorption assessment at higher temperatures, sorption coefficient (*K*_F_) and the octanol–water partition coefficient (log *K*_ow_) relationship (PDF)
