# Supplementary material for: Adsorptive Removal of PFAS from Aqueous Solutions Using GAC, PAC and Ball-Milled Colloidal Activated Carbon: Characterizing Efficiency, Kinetics, and Mechanisms
Source: ACS ES T Water. 2025 Nov 3;5(11):6554–66. doi: 10.1021/acsestwater.5c00641 (PMC12624724; doi:10.1021/acsestwater.5c00641)
Supplement: Supplementary file 1 [file ew5c00641_si_001.pdf]

Appendix A

Supplementary Information

Adsorptive Removal of PFAS from Aqueous Solutions Using GAC, PAC and Ball-Milled Colloidal Activated Carbon: Characterizing Efficiency, Kinetics, and Mechanisms

Mahlet M. Kebede[1], Md Abdullah Al Masud[1], Sarah Ortbal[1], Won Sik Shin[2], Mesfin M. Mekonnen[1], T. Prabhakar Clement[1], and Leigh G. Terry[1*]

[1]Department of Civil, Construction, and Environmental Engineering, University of Alabama, Tuscaloosa, Alabama 35487, United States (Mahlet M Kebede, M.S.: mkebede@crimson.ua.edu; M. A. A. Masud, Ph.D.: mmasud1@ua.edu; Sarah K. Ortbal, M.S.: skortbal@crimson.ua.edu; Mesfin M. Mekonnen, Ph.D.:mesfin.mekonnen@ua.edu; T.P. Clement, Ph.D.: pclement@ua.edu) ; L.G. Terry, Ph.D.: leigh.terry@ua.edu)

[2]School of Architecture, Civil, Environmental and Energy Engineering, Kyungpook National University, Daegu 41566, Republic of Korea (W.S. Shin, Ph.D.: wshin@knu.ac.kr)

***Correspondence to:** Leigh G. Terry, Department of Civil, Construction, and Environmental Engineering, University of Alabama, Tuscaloosa, Alabama 35487, United States.

Phone: (205) 348-1580; **E-mail:** leigh.terry@ua.edu

The supplementary information includes (14 pages):

Text: S1-S5, pp. 2-6

Tables: S1–S9, pp. 7-10

Figures: **S1-S5,** pp. 11-13

References: pp. 14-15

Text S1. Physicochemical characterization of CAC_{BM}

The BET surface area (A_{BET}) of CAC_{BM} was determined by applying the Brunauer–Emmett–Teller (BET) model to nitrogen (N_2) adsorption–desorption isotherms. Pore size distribution was assesed using the Barrett–Joyner–Halenda (BJH) adsorption model with a surface area analyzer (Quantachrome, Autosorb-iQ & Quadrasorb Si, USA; Nova 2000, USA). Surface morphology was characterized using field emission scanning electron microscopy (FE-SEM, Hitachi Corp., S4300, Japan). Additionally, particle size distribution of CAC_{BM} was determined using a dynamic light scattering (DLS) analysis (Malvern Panalytical Ltd., Mastersizer 3000, UK). Fourier transform infrared (FTIR) spectroscopy (AVATAR, Thermo Nicolet spectrophotometer) was used to assess the functional groups and covalent bonds in CAC_{BM} and PFAS before and after adsorption experiments. The point of zero charge (pH_{pzc}), defined as the pH at which the net surface charge of the material is zero, was measured following the method described by Appel et al. (2003)[1], as briefly described here.

Text S2. Determination of point of zero charge (pH_{PZC})

The point of zero charge (pH_{PZC}) was determined using the method by Appel et al. (2003)[1] . Briefly, 0.1 g of the ball-milled colloidal activated carbon (CAC_{BM}) was added to 50 mL conical tubes (Polypropylene). Then, 10 mL of 0.1, 0.01, and 0.001 M NaCl were added as electrolyte solutions. The initial pH of the solutions was adjusted from 3 to 12 by adding 0.1 M HCl or 0.1 M NaOH. The conical tubes were filled with 25 mL of DDI water. The samples were mixed in a shaker for 7 d at 200 rpm and 25 °C to achieve equilibrium. The final pH values of the solutions were measured at the end of the incubation period using a pH meter (Orion 3 Star pH Benchtop, Thermo Scientific, USA). Amounts of H^+ and OH^- adsorbed by CAC_{BM} were determined by

subtracting the amount of 0.1 M HCl and 0.1 M NaOH required to bring 10 mL of electrolyte plus 15 mL of DDI water (no CAC_{BM}) to the initial pH.

Text S3. Determination of acidic and alkaline sites of CAC_{BM}

The concentration of acidic and alkaline sites of CAC_{BM} was determined by pH drift and the Boehm titration method[2,3], respectively, using a Metrohm automatic titrator (877 Titrino plus, Metrohm, Switzerland) and continuous stirring at 25 °C. Briefly, 0.1 g of CAC_{BM} was added to 40 mL of 50 mM Boehm reactant solution (i.e., NaOH, Na_2CO_3 or $NaHCO_3$) in the Boehm titration chamber. The samples were mixed for 72 h followed by filtration to remove the carbon materials. Then, 10 mL of NaOH or $NaHCO_3$, and 5 mL of Na_2CO_3 aliquots were taken from the samples using a pipette. These solutions were acidified by the addition of 20 mL of 0.05 M HCl solution and back titrated with 0.05 M NaOH solution. A control experiment without CAC_{BM} was also conducted to determine the effect of glassware surface adsorption on the titration. All experiments were run in triplicate.

Text S4. Adsorption kinetics and isotherm models

The pseudo-first-order model (PFOM) (**Eq. S1**) describes solid-liquid adsorption systems in which the physical adsorption of the adsorbate is notably more dominant than any possible chemical adsorption. The PFOM equation is expressed as follows[4] :

$$q = q_e(1 - e^{-k_1 t}) \quad \text{(S1)}$$

where q_e and q are the adsorption capacity at equilibrium and at any time of the adsorption (mg g^{-1}) respectively, k_1 is the PFOM rate constant (min^{-1}) and t is time (min).

In contrast, the pseudo-second-order model (PSOM) (**Eq. S2**) suggests that chemical adsorption of the adsorbate onto the surface of the adsorbent is occurring. Its equation is expressed as[5] :

$$q = \frac{{q_e}^2 k_2 t}{1 + q_e k_2 t} \quad \text{(S2)}$$

where k_2 is the PSOM rate constant (g mg^{-1} min^{-1}).

The pseudo-first-order and pseudo-second-order models were applied to analyze the adsorption kinetics in this study. It should be noted that these models are empirical, and their applicability does not definitively distinguish between physisorption and chemisorption mechanisms

The intraparticle diffusion model (IPDM) describes the diffusion of adsorbates within the adsorbent structure (**Eq. S3**)[6]. It is mostly used to identify the rate-limiting mechanisms in adsorption processes, and it is expressed as:

$$q_t = k_d t^{0.5} + C_e \quad \text{(S3)}$$

where k_d is the IPDM rate constant (mg g^{-1} min^{-1}) and C_e is a constant of the experiment (mg g^{-1}).

The Freundlich isotherm model (**Eq. 4**)[7] describes adsorption on a heterogeneous surface, where adsorption sites vary in affinity, with the most energetically favorable sites being occupied preferentially. The nonlinear Freundlich model was used to fit the experimental data in this study, as follows:

$$q = K_F {C_e}^{1/n}$$

(S4)

where K_F is the Freundlich adsorption coefficient [(μmol g^{-1}) (μmol L^{-1})$^{-(1/n)}$] and 1/n (–) is the Freundlich exponent.

Since the units of K_F depend on Freundlich's exponent (1/n) nonlinearly, partitioning coefficients K_d (the ratio of q to C) were estimated to compare the adsorption affinities across treatments.

$$K_d = K_F C^{((1/n)-1)} \quad \text{(S5)}$$

The Langmuir model (**Eq. S6**)[8] assumes monolayer adsorption onto a homogeneous surface with a finite number of adsorption sites, each possessing equal affinity for the adsorbate. The Langmuir model was applied to fit the experimental data, as expressed by the following equation:

$$q = \frac{q_{mL} b_L C}{1+b_L C} \quad \text{(S6)}$$

where q_{mL} (μmol g^{-1}) and b_L (L μmol^{-1}) are the Langmuir model parameters that represent maximum adsorption capacity and the site energy factor, respectively.

Polanyi-Dubinin-Manes (PDM) model was used to fit adsorption data[9] :

$$q = q_{max}\, exp\left\{-\alpha \left(\frac{\varepsilon}{V_m}\right)^d\right\}_{max} \quad \text{(S7)}$$

$$\varepsilon = \mathrm{RT} \ln\left(\frac{S_w}{C}\right) \quad \text{(S8)}$$

where q_{max} is the maximum adsorption capacity (mmol g^{-1}), ε is Polanyi potential (= RT ln(S/C), J mol^{-1}), R is the ideal gas constant (= 8.314 J mol^{-1} K^{-1}), T is the temperature (K), S_w is the compound's solubility (mmol L^{-1}), V_m is molar volume the solute (mL mol^{-1}), respectively, and α $[(mL)^{d+1} (mol\ J^d)^{-1}]$ and *d,* are the fitting parameters[10]

Text S5. PFAS analysis

PFAS concentration was analyzed using Agilent 1290 Infinity II liquid chromatography coupled to 6465A triple quadrupole mass spectrometer system (Ultivo LC/TQ Agilent Inc) with Agilent jet stream technology ion source (AJS). Zorbax Eclipse Plus-C18 column (2.1 × 100 mm, 1.8 μm, Agilent Technologies, USA) and short delay column (4.6 × 30 mm) were heated to 40 °C for chromatographic separation. The mobile phase compositions were mixtures of ammonium

acetate (2 mM) with acetonitrile (ACN): water (5%:95%, v/v) for mobile phase A and ACN (100%, v/v) for mobile phase B. Sonication was used to degas mobile phase A. A constant flow rate of 0.4 mL min^{-1} with 5 µL injection volume was set for the gradient elution (**Table S2**). The stock native and mass labeled PFAS solutions were diluted in DI water to a concentration of 100 ng mL^{-1}. The primary and stock solutions were stored at less than 4 °C

The mass spectrometer was operated in negative mode with a nebulizer nitrogen gas pressure of 20 psi, capillary voltage +2.5 kV, sheath gas flow rate of 10 L min^{-1}, and sheath gas temperature of 355°C. The optimized transitions of precursor ions to product ions and collision energies are summarized in **Table S3**. Data acquisition and processing were performed using Agilent MassHunter Acquisition (version 1.2) and Agilent MassHunter Quantitative (version 12.0) software programs, respectively. The target analytes were quantified using isotopic dilution methods including an internal standard corrected five-point calibration curve (0.5–100 ng mL^{-1}). The linear regression fitting of the calibration curves gave $R^2 > 0.99$ for all batches of analysis. QC samples spiked with target PFAS method blanks, and instrument blanks were analyzed in each sample batch and injected periodically to monitor instrument performance, sample carryover, and other laboratory contamination.

Table S1. A brief summary of key previous articles discussing the adsorption of various contaminants using different types of colloidal and ball-milled activated carbon

Study	CAC type	Contaminant	Focus
Mole et al. (2022)[11]	Intraplex®	PFAS	Groundwater chemistry and adsorption of short chins PFAS on CAC
Hakimabadi et al. (2023)[12]	PlumeStop® and another polymer free CAC supplied by Regenesis	PFAS	Factors affecting adsorption of PFAS on CAC
Niarchos et al. (2023)[13]	PlumeStop®	PFAS	Competitive sorption of PFAS on CAC
Jiang et al. (2025)[14]	Intraplex®	PFAS	Aging of CAC on PFAS adsorption
Bakkaloglu et al. (2021)[15]	Superfine pulverized AC	Carbamazepine	Removal of Carbamzepine from natural source water
Bonvin et al. (2016)[16]	Superfine PAC	Micropollutatnt	Removal of micropollutants from wastewater
Li et al. (2024) [17]	Ball-milled PAC	Organic pesticides	Investigated the removal efficiencies of six pesticides
This study	Ball-milled CAC (non-proprietary CAC)	PFAS	side-by-side comparison of GAC, PAC, and ball-milled CAC under controlled laboratory conditions, with a focus on adsorption kinetics and the influence of water chemistry

Table S2. Physicochemical properties of the PFAS used in this study

PFAS	Formula	Molecular weight (g mol^{-1})	log K_{ow}	Chain length	Molecular length (nm)	pKa
Perfluorooctanic acid (PFOA)	$C_8HF_{15}O_2$	414.07	4.59	8	1.11	2.8
Perfluorooctane sulfonic acid (PFOS)	$C_8HF_{17}SO_3$	538.22	5.26	8	1.33	-3.27
Perfluorobutanic acid (PFBA)	$C_4HF_7O_2$	214.04	2.32	4	0.6	0.4
Perfluorobutane sulfonic acid (PFBS)	$C_4HF_9SO_3$	338.19	2.73	4	0.81	0.14

Table S3. Gradient program used in Liquid Chromatography analysis

Parameter	Value	
Gradient	Time (min)	Percentage of Mobile Phase B
	0	2
	0.2	2
	10	95
	10.01	2
Stop time	12.2 min	
Post time	4 min	

Table S4. Mass spectrometer optimized transitions and parameters

Compound Name	Transition	Precursor Ion	Product Ion	Collision Energy	Fragmentor
PFOA	413.0 -> 369.0	413.0	369.0	4	69
PFOA	413.0 -> 169.0	413.0	169.0	12	69
PFOS	498.9 -> 80.0	498.9	80.0	50	100
PFOS	498.9 -> 99.0	498.9	99.0	50	100
PFBA	213.0 -> 168.9	213.0	168.9	8	60
PFBS	298.9 -> 80.0	298.9	80.0	45	100
PFBS	298.9 -> 98.9	298.9	98.9	29	100
$^{13}C_8$-PFOA	421.0 -> 376.0	421.0	376.0	4	69
$^{13}C_8$-PFOA	421.0 -> 172.0	421.0	172.0	20	72
$^{13}C_8$-PFOS	507.0 -> 80.0	507.0	80.0	50	100
$^{13}C_8$-PFOS	507.0 -> 99.0	507.0	99.0	52	148
$^{13}C_8$-PFBA	217.0 -> 172.0	217.0	172.0	8	60
$^{13}C_3$-PFBS	302.0 -> 99.0	302.0	99.0	32	130
$^{13}C_3$-PFBS	302.0 -> 80.0	302.0	80.0	44	130

Table S5. Physicochemical characteristics of GAC, PAC, and CAC_{BM}

**Characteristics of Norit® 830W**	**GAC**	**PAC**	**CAC_{BM}**
pH	8.94	7.48	6.55
pH_{pzc}	10.03	8.42	5.90
BET surface area (A_{BET}, $m^2\ g^{-1}$)	716.51	729.94	968.59
Micropore volume, V_{micro} (cm^3g^{-1})	0.166	0.180	0.250
Mesopore volume, V_{meso} (cm^3g^{-1})	0.038	0.043	0.089
Total pore volume (cm^3g^{-1})	0.204	0.223	0.339
Pore size (Å) (nm)	4.32	3.88	3.32
Particle size, d_{50} (μm)	ND[a]	59.23	0.318

[a]t-Plot micropore volume; [b]Adsorption average pore diameter [c]Not Determined

Table S6. Composition of surface functional groups of PAC and CAC_{BM}.

**Carbon catalyst**	**pH**	**Carboxyl group (μmol g^{-1})**	**Phenol group (μmol g^{-1})**	**Lactone group (μmol g^{-1})**	**Acidity (μmol g^{-1})**	**Alkalinity (μmol g^{-1})**
PAC	7.48	42.23	53.02	21.23	120.60	117.56
CAC_{BM}	6.71	50.98	64.93	15.67	131.58	105.39

Note. The pH of solvent (DI water) = 6.43

Table S7. Summary of key physicochemical properties and pH-dependent adsorption behavior of selected PFAS on CAC_{BM}.

**Parameter**	**PFOA**	**PFOS**	**PFBA**	**PFBS**
Functional group	–COOH	$–SO_3H$	–COOH	$–SO_3H$
pKa	~2.8	<1	~2.5	<1
Charge at pH 3	Anionic (mostly)	Anionic	Anionic (mostly)	Anionic
Max adsorption pH	3.0	6.2	3.0	3.0
Main interaction	Electrostatic attraction	Hydrophobic attraction	Electrostatic attraction	Electrostatic attraction
Effect of sorbent (pHpzc = 5.9)	Positive surface at pH < 5.9, attracting anions	Neutral-to-negative at pH 6.0	Positive surface at pH < 5.9, attracting anions	Positive surface at pH < 5.9, attracting anions

Table S8. Polanyi−Dubinin−Manes (PDM) isotherm model parameters for adsorption of PFAS onto GAC and CAC_{BM}

Compound	Material	q_{max} (µmol g^{-1})	α [(mL)$^{d+1}$ (mol J^{d})$^{-1}$]	R^2
PFOA	GAC	66.0	4.4×10^{-17}	0.995
	CAC_{BM}	220.7	3.1×10^{-15}	0.998
PFOS	GAC	101.0	4.3×10^{-11}	0.991
	CAC_{BM}	371.8	2.1×10^{-1}	0.992
PFBS	GAC	228.6	6.2×10^{-21}	0.982
	CAC_{BM}	494.0	4.1×10^{-7}	0.986

Table S9. Key FTIR Peaks and Corresponding Functional Groups

Wavenumber (cm^{-1})	Assigned Functional Group	Interpretation	Reference
~3241–3243	O–H stretching (hydroxyl groups)	Indicates the presence of surface hydroxyl groups or adsorbed water. These are commonly associated with –OH from alcohols, phenols, or carboxylic acids.	Liu et. al (2017)[18]
~2172–2179	C≡C or C≡N stretching (alkynes or nitriles) *(possibly noise or impurities)*	These peaks are uncommon in activated carbon and may be attributed to adsorbed PFAS-related artifacts or weak overtone bands.	Fedoseeva et al. (2020)[19]
~2142–2146	Possible –CF stretching or atmospheric CO_2 overtones	Could relate to fluorinated species interaction or weak carbon-related overtone bands.	Azzolina-Jury et al. (2017)[20]
~1576–1591	C=C stretching in aromatic structures	Confirms the presence of graphitic/aromatic domains in the activated carbon structure.	Tipplook et al. (2024)[21] Zhang et al. (2024)[22]
~1079–1086	C–O stretching (ethers, alcohols, carboxylic acids)	Suggests oxygen-containing surface functionalities like carboxylic or phenolic groups.	Nasrollahpour et al. (2024)[23]
~800 and 464	Out-of-plane bending (aromatic C–H or lattice vibrations)	Often associated with the carbon backbone and possibly some mineral residues.	Wang et al. (2024)[24]

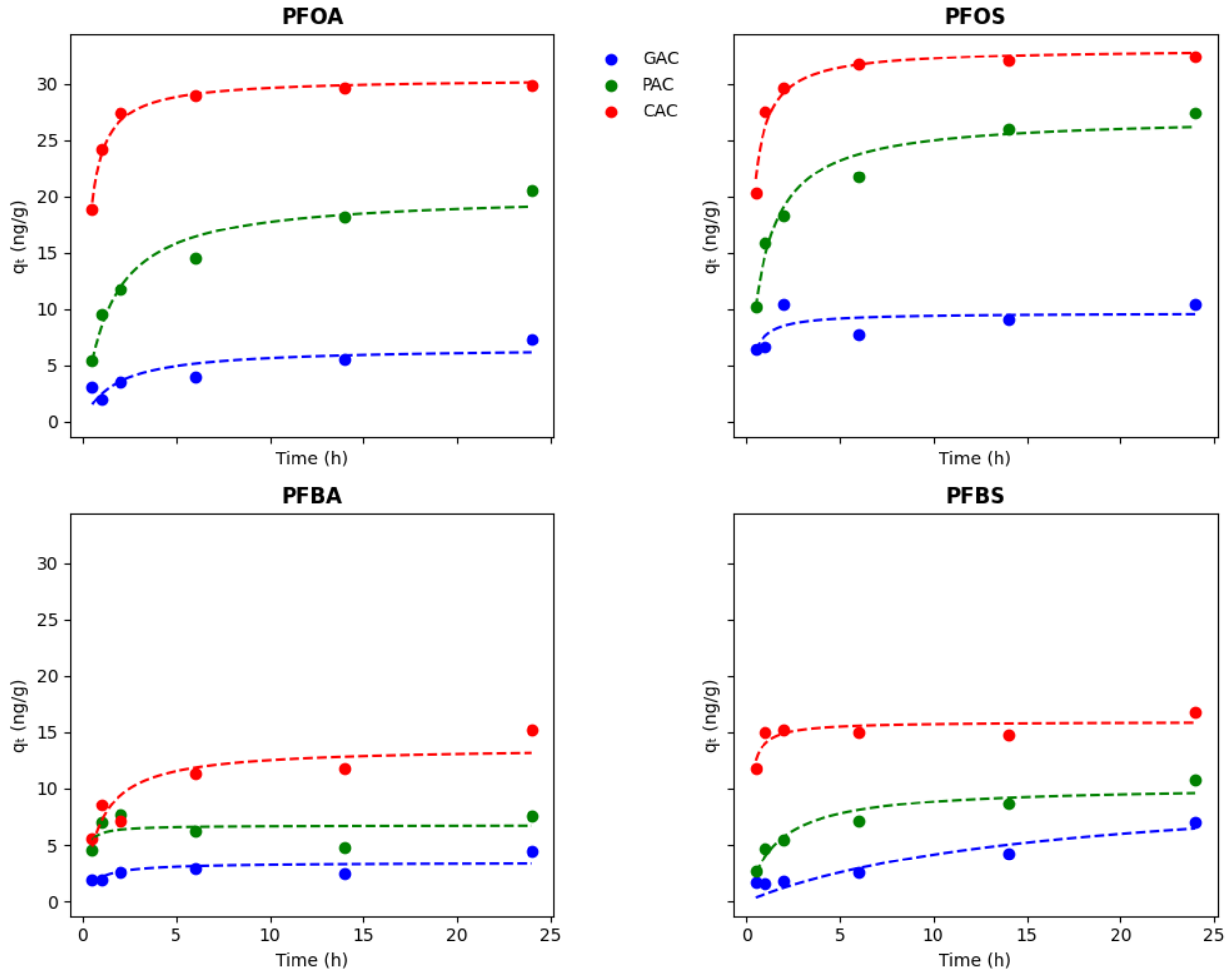

Fig. S1. Adsorption kinetics of PFOA, PFOS, PFBA, and PFBS onto GAC, PAC, and ball-milled colloidal activated carbon (CAC_{BM}). Experimental data are shown as points, and fitted pseudo-second-order (PSO) model curves are shown as dashed lines. Experimental conditions: $[CAC_{BM}]_0$ = 30 mg L^{-1}, initial PFAS concentration = 1000 ng mL^{-1}, pH = 6.2 (unadjusted), T = 22 °C (room temperature).

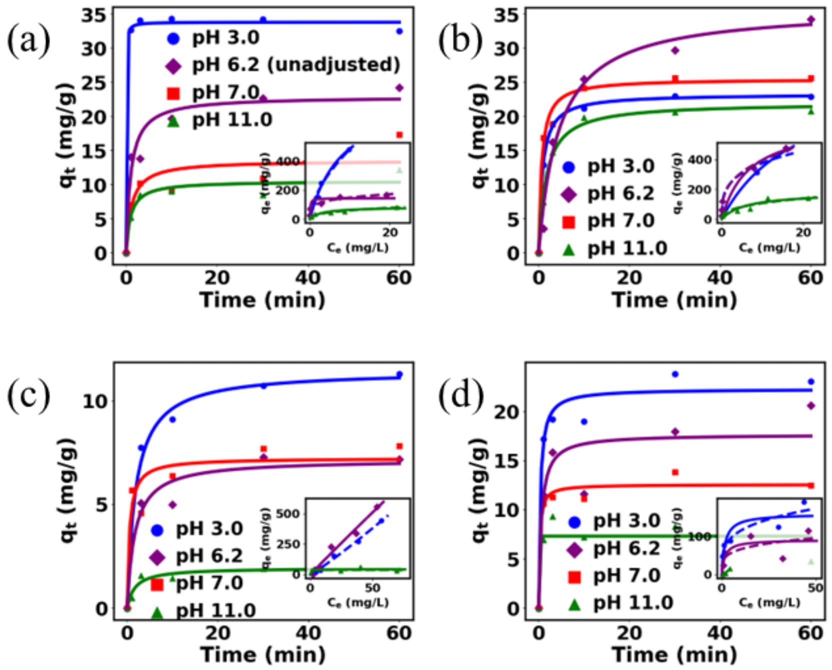

Fig S2. Comparison of adsorption kinetics at different pH values for **(a)** PFOA, **(b)** PFOS, **(c)** PFBA, and **(d)** PFBS. Insets show Langmuir (solid line) and Freundlich (dashed line) isotherm fits. Experimental conditions: $[PFAS]_0 = 1.0$ mg L^{-1}, $[CAC_{BM}]_0 = 30$ mg L^{-1}, Temperature = 22 °C (room temperature), contact time = 1.0 h, pH = 6.2 (unadjusted).

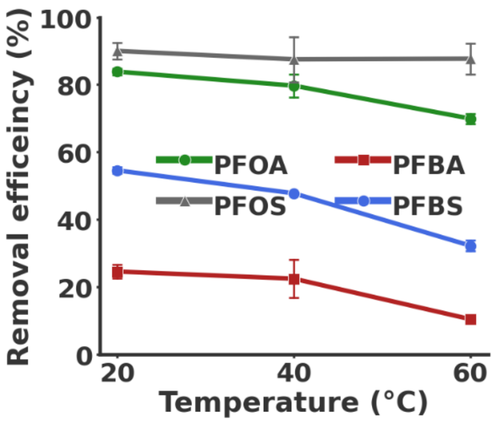

Figure S3. Removal efficiency of PFAS in 1-hr batch experiments at different temperatures (20 °C to 60 °C)

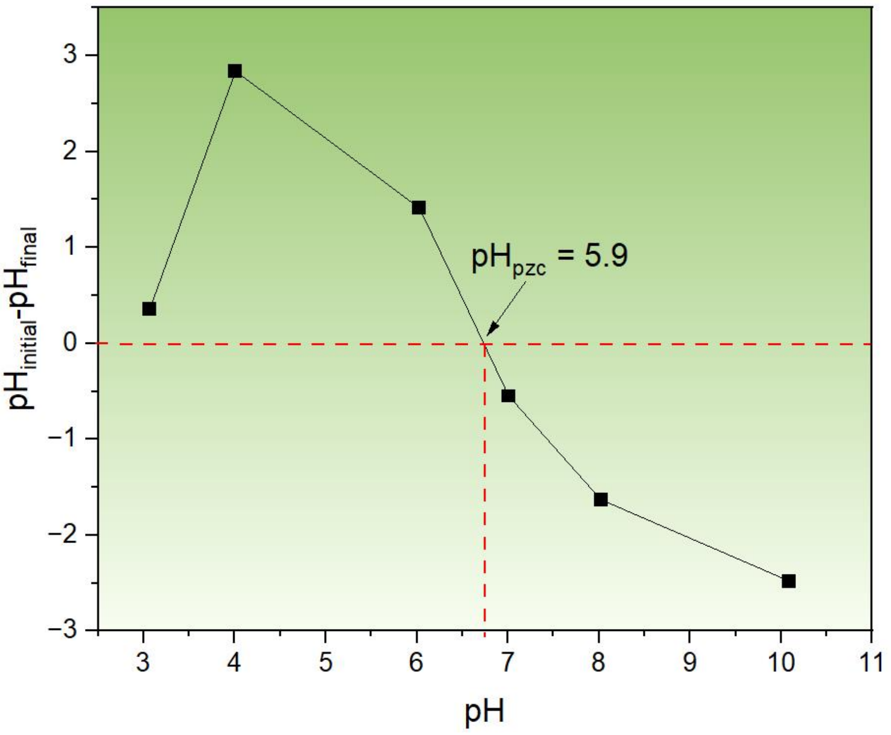

Fig. S4. The pH$_{PZC}$ of CAC$_{BM}$.

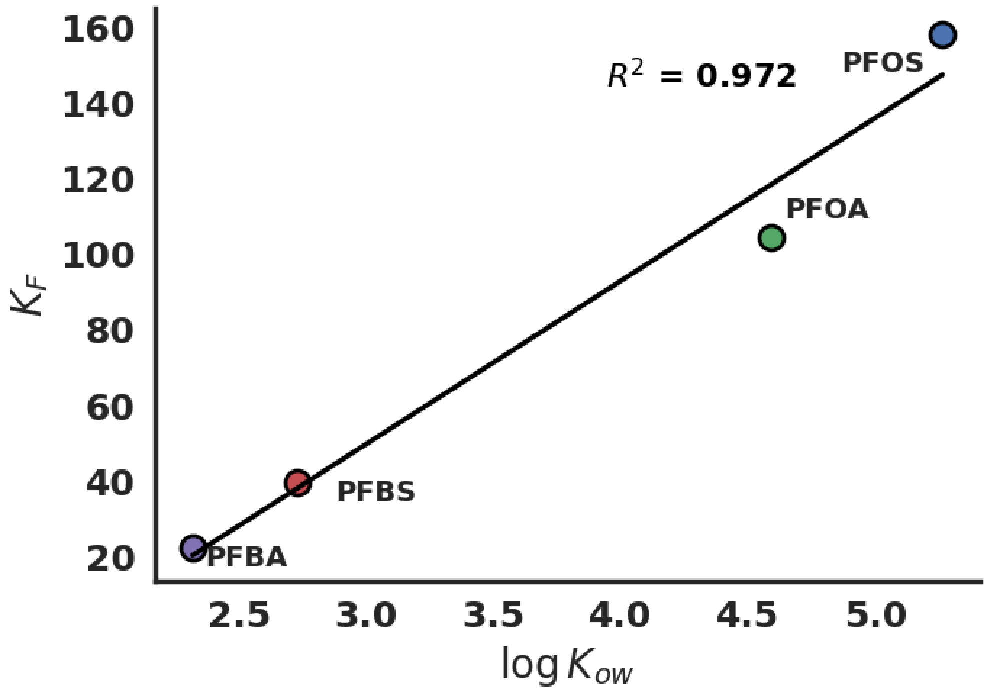

Fig. S5. Plot of sorption coefficient (K_F) and the octanol-water partition coefficient (log K_{ow})

References

(1) Appel, C.; Ma, L. Q.; Dean Rhue, R.; Kennelley, E. Point of Zero Charge Determination in Soils and Minerals via Traditional Methods and Detection of Electroacoustic Mobility. *Geoderma* **2003**, *113* (1), 77–93. https://doi.org/10.1016/S0016-7061(02)00316-6.

(2) Burgess, A. E.; Davidson, J. C. A Kinetic–Equilibrium Study of a Triiodide Concentration Maximum Formed by the Persulfate–Iodide Reaction. *J. Chem. Educ.* **2012**, *89* (6), 814–816. https://doi.org/10.1021/ed200055t.

(3) Schönherr, J.; Buchheim, J. R.; Scholz, P.; Adelhelm, P. Boehm Titration Revisited (Part I): Practical Aspects for Achieving a High Precision in Quantifying Oxygen-Containing Surface Groups on Carbon Materials. *C* **2018**, *4* (2), 21. https://doi.org/10.3390/c4020021.

(4) Lagergren, S. Zur Theorie Der Sogenannten Adsorption Geloster Stoffe. *Kungliga svenska vetenskapsakademiens. Handlingar* **1898**, *24*, 1–39.

(5) Ho, Y. S.; McKay, G. Pseudo-Second Order Model for Sorption Processes. *Process Biochemistry* **1999**, *34* (5), 451–465. https://doi.org/10.1016/S0032-9592(98)00112-5.

(6) Morris, J. C.; Weber JR, W. J. Removal of Biologically-Resistant Pollutants from Waste Waters by Adsorption. In *Advances in water pollution research*; Elsevier, 1964; pp 231–266.

(7) Freundlich, H. *Kapillarchemie: Eine Darstellung Der Chemie Der Kolloide Und Verwandter Gebiete*; akademische Verlagsgesellschaft, 1922.

(8) Langmuir, I. The Adsorption of Gases on Plane Surfaces of Glass, Mica and Platinum. *Journal of the American Chemical society* **1918**, *40* (9), 1361–1403.

(9) Allen-King, R. M.; Grathwohl, P.; Ball, W. P. New Modeling Paradigms for the Sorption of Hydrophobic Organic Chemicals to Heterogeneous Carbonaceous Matter in Soils, Sediments, and Rocks. *Advances in Water Resources* **2002**, *25* (8), 985–1016. https://doi.org/10.1016/S0309-1708(02)00045-3.

(10) Kleineidam, S.; Schüth, C.; Grathwohl, P. Solubility-Normalized Combined Adsorption-Partitioning Sorption Isotherms for Organic Pollutants. *Environ. Sci. Technol.* **2002**, *36* (21), 4689–4697. https://doi.org/10.1021/es010293b.

(11) Molé, R. A.; Velosa, A. C.; Carey, G. R.; Liu, X.; Li, G.; Fan, D.; Danko, A.; Lowry, G. V. Groundwater Solutes Influence the Adsorption of Short-Chain Perfluoroalkyl Acids (PFAA) to Colloidal Activated Carbon and Impact Performance for in Situ Groundwater Remediation. *Journal of Hazardous Materials* **2024**, *474*, 134746. https://doi.org/10.1016/j.jhazmat.2024.134746.

(12) Hakimabadi, S. G.; Taylor, A.; Pham, A. L.-T. Factors Affecting the Adsorption of Per- and Polyfluoroalkyl Substances (PFAS) by Colloidal Activated Carbon. *Water Research* **2023**, *242*, 120212. https://doi.org/10.1016/j.watres.2023.120212.

(13) Niarchos, G.; Georgii, L.; Ahrens, L.; Kleja, D. B.; Fagerlund, F. A Systematic Study of the Competitive Sorption of Per- and Polyfluoroalkyl Substances (PFAS) on Colloidal Activated Carbon. *Ecotoxicology and Environmental Safety* **2023**, *264*, 115408. https://doi.org/10.1016/j.ecoenv.2023.115408.

(14) Jiang, L.; Chen, X.; Carey, G. R.; Liu, X.; Lowry, G. V.; Fan, D.; Danko, A.; Li, G. Effects of Physical and Chemical Aging of Colloidal Activated Carbon on the Adsorption of Per- and Polyfluoroalkyl Substances. *Environ. Sci. Technol.* **2025**, *59* (7), 3691–3702. https://doi.org/10.1021/acs.est.4c07958.

(15) Bakkaloglu, S.; Ersan, M.; Karanfil, T.; Apul, O. G. Effect of Superfine Pulverization of Powdered Activated Carbon on Adsorption of Carbamazepine in Natural Source Waters.

Science of The Total Environment **2021**, *793*, 148473. https://doi.org/10.1016/j.scitotenv.2021.148473.
(16) Bonvin, F.; Jost, L.; Randin, L.; Bonvin, E.; Kohn, T. Super-Fine Powdered Activated Carbon (SPAC) for Efficient Removal of Micropollutants from Wastewater Treatment Plant Effluent. *Water Research* **2016**, *90*, 90–99. https://doi.org/10.1016/j.watres.2015.12.001.
(17) Li, W.; Dong, C.; Hao, Z.; Wu, X.; Ding, D.; Duan, J. The Effectiveness and Feasibility of Ball-Milled Powdered Activated Carbon (BPAC) for Removal of Organic Pesticides in Conventional Drinking Water Treatment Process. *Chemosphere* **2024**, *359*, 142229. https://doi.org/10.1016/j.chemosphere.2024.142229.
(18) Liu, Y.; Liu, X.; Dong, W.; Zhang, L.; Kong, Q.; Wang, W. Efficient Adsorption of Sulfamethazine onto Modified Activated Carbon: A Plausible Adsorption Mechanism. *Sci Rep* **2017**, *7* (1), 12437. https://doi.org/10.1038/s41598-017-12805-6.
(19) Fedoseeva, Y. V.; Lobiak, E. V.; Shlyakhova, E. V.; Kovalenko, K. A.; Kuznetsova, V. R.; Vorfolomeeva, A. A.; Grebenkina, M. A.; Nishchakova, A. D.; Makarova, A. A.; Bulusheva, L. G.; Okotrub, A. V. Hydrothermal Activation of Porous Nitrogen-Doped Carbon Materials for Electrochemical Capacitors and Sodium-Ion Batteries. *Nanomaterials* **2020**, *10* (11), 2163. https://doi.org/10.3390/nano10112163.
(20) Azzolina-Jury, F.; Thibault-Starzyk, F. Mechanism of Low Pressure Plasma-Assisted CO2 Hydrogenation Over Ni-USY by Microsecond Time-Resolved FTIR Spectroscopy. *Top Catal* **2017**, *60* (19), 1709–1721. https://doi.org/10.1007/s11244-017-0849-2.
(21) Tipplook, M.; Hisama, K.; Koyama, M.; Fujisawa, K.; Hayashi, F.; Sudare, T.; Teshima, K. Cation-Doped Nanocarbons for Enhanced Perfluoroalkyl Substance Removal: Exotic Bottom-Up Solution Plasma Synthesis and Characterization. *ACS Appl. Mater. Interfaces* **2024**, *16* (45), 61832–61845. https://doi.org/10.1021/acsami.4c08925.
(22) Zhang, R.; Ren, Z.; Bergmann, U.; Uwayezu, J. N.; Carabante, I.; Kumpiene, J.; Lejon, T.; Levakov, I.; Rytwo, G.; Leiviskä, T. Removal of Per- and Polyfluoroalkyl Substances (PFAS) from Water Using Magnetic Cetyltrimethylammonium Bromide (CTAB)-Modified Pine Bark. *Journal of Environmental Chemical Engineering* **2024**, *12* (5), 114006. https://doi.org/10.1016/j.jece.2024.114006.
(23) Nasrollahpour, S.; Tanhadoust, A.; Pulicharla, R.; Brar, S. K. Long-Chain Perfluoroalkyl Carboxylic Acids Removal by Biochar: Experimental Study and Uncertainty Based Data-Driven Predictive Model. *iScience* **2024**, *27* (11).
(24) Wang, X.; He, X.; Wang, X. FTIR Analysis of the Functional Group Composition of Coal Tar Residue Extracts and Extractive Residues. *Applied Sciences* **2023**, *13* (8), 5162. https://doi.org/10.3390/app13085162.
